# Effect of Korean Advanced Life Support Education on Non-Technical and Technical Skills of Nursing Students: A Pilot Study

**DOI:** 10.3390/healthcare9101253

**Published:** 2021-09-23

**Authors:** Yon Hee Seo, Kyong Ah Cho

**Affiliations:** 1 Department of Nursing, Yeoju Institute of Technology, Yeoju 12652, Korea; yseo017@naver.com; 2 Department of Nursing, Gwangju University, Gwangju 61743, Korea

**Keywords:** basic life support, cardiac arrest, Korean Advanced Life Support, non-technical skills, nursing student

## Abstract

This study aimed to investigate the effect of the Korean Advanced Life Support (KALS) education program on the non-technical skills and technical skills of nursing students. This one-group pretest–posttest experimental study included 46 participants who were fourth year nursing students at Shinsung University located in Dangjin-si, Chungcheongnam-do Province, Republic of Korea. Data were collected in April 2021 and analyzed via SPSS/WIN 25.0, using a paired samples *t*-test. The current study results report a significant improvement in the non-technical skills from 30.58 to 47.16 points (*t* = −5.892, *p* < 0.001). Furthermore, KALS training improved communication confidence from 23.45 to 35.77 points (*t* = −6.563, *p* < 0.001), critical thinking tendency from 96.71 to 107.16 points (*t* = −3.352, *p* = 0.002), and self-efficacy in performing cardiopulmonary resuscitation from 33.56 to 49.81 points (*t* = −13.242, *p* < 0.001). Lastly, technical skills also improved from 18.35 to 27.94 points (*t* = −28.439, *p* < 0.001). Therefore, the findings indicate that KALS education was effective in improving the non-technical and technical skills of these nursing students. However, this study did not analyze the effect of the stress level experienced by the study participants in emergency situations on their non-technical and technical skill performance. Thus, future studies should verify the effect of external stressors, caused by unpredictable emergencies, on non-technical and technical skill performance.

## 1. Introduction

There is no significant difference between the survival rate and prognosis of cardiac arrest inpatients and outpatients [1]. This finding is influenced by patients’ health status, along with various other factors such as the level of resuscitation imposed by medical staff, delayed treatment adherence duration, the decision regarding patient monitoring before cardiac arrest, and integrated treatment after resuscitation [1,2]. The initial response to cardiac arrest within a hospital is of utmost importance since brain death occurs 4–5 min after a cardiac arrest [3]. Therefore, prompt and accurate treatment responses by medical staff should be emphasized. Nurses are particularly more likely to first witness a cardiac arrest or emergency situations, due to their 24 h proximity to the patient to provide care [4]. Thus, a nurse’s ability to perform cardiopulmonary resuscitation (CPR) can be an important factor in improving the survival rate of patients with cardiac arrest [5].

Nurses perform basic resuscitation—implementing emergency drugs and specialized airway maintenance—for most cases of cardiac arrest within hospitals. Moreover, the need for administering defibrillation and emergency drugs varies according to the electrocardiogram (ECG) rhythm analysis during treatment. Therefore, it is necessary to understand cardiac arrest rhythm analysis for effectively performing professional resuscitation and CPR [6] while maintaining leadership and teamwork to facilitate coordination of activities within the team, such that each team can simultaneously perform their respective roles. For this purpose, it is recommended to provide education on non-technical skills including patient and situation management between various team members [7]. However, the existing curriculum of the Korean University of Nursing does not include any mandates for emergency cardiological education, such as CPR. Currently, emergency education is limited. Nursing colleges either arbitrarily teach some subjects such as basic, emergency, and adult nursing, or they merely aim to satisfy the learning outcomes prescribed by the Korea Institute of Nursing Education and Evaluation. Thus, the current education focuses only on teaching technical skills to provide training for single-rescuer and two-rescuer resuscitations in basic life support (BLS) of the American Heart Association (AHA). Thus, the non-technical skill education recommended by the Korea Association of Cardiopulmonary Resuscitation is insufficient.

Non-technical skills refer to the cognitive and social skills that complement technical skills, in order to ensure safe and effective work performance, and include leadership, teamwork, decision making, and situational awareness (i.e., the ability to understand team roles and responsibilities as an effective medical team and implement information for conflict resolution) [8,9]. Therefore, these are foundational elements for enabling improvements in patient prognosis and outcomes [10]. However, the lack of non-technical skills is considered to be a major cause of unavoidable errors during CPR within the healthcare field. Thus, it is essential to improve non-technical skill education to reduce these errors and improve patient outcomes [11].

Anderson et al. [12] conducted a study on CPR-related non-technical skills and subsequently encompassed five domains (CPR leadership, communication, mutual performance monitoring, compliance with standards and guidelines, and business management). Similarly, Walker et al. [13] elucidated six domains (communication, collaboration, coordination, leadership, monitoring, and decision making), while Cooper et al. [14] presented three elements—leadership, teamwork, and work management—to emphasize the importance of non-technical skills. However, in Korea, there are no clear existing guidelines for non-technical skill training within CPR-related situations. The Korean Cardiopulmonary Resuscitation Association introduced the Advanced Cardiovascular Life Support (ACLS) provider course, developed by the AHA, for Korean healthcare workers to provide information on the cultural differences, duration, and contents of ACLS. The Korean Advanced Life Support (KALS) program was developed to suit domestic conditions and account for the differences between non-technical and technical skills [7]. It comprises a cardiac arrest simulation training course modeled on a medical emergency team, and it classifies cases by ward, patient type, and disease to educate students regarding the most common cardiac arrest cases observed in Korean hospitals. Therefore, this study aimed to examine the effect of KALS practice education on the non-technical and technical skill performance of nursing students. This study will provide basic data for developing an effective educational method that can enhance nursing students’ practical ability to cope with emergency situations.

## 2. Materials and Methods

### 2.1. Research Design

This study adopted a one-group pretest–posttest experimental design to investigate the effect of KALS education on nursing students’ performance of non-technical skills and technical skills.

### 2.2. Participants

This study employed convenient sampling to select 4th year nursing students at Shinsung University, located in Dangjin-si, Chungcheongnam-do Province, Republic of Korea. Those who understood the purpose of this study and provided consent were recruited for participation. The sample size was determined by performing a paired samples *t*-test using the G*Power software (version 3.1.7, Heinrich-Heine-University, Germany) to obtain the optimal significance level (α < 0.05) and power (1-β). The output suggested a minimum sample size of 34 participants to ensure a power of 0.80 and an effect size (d) of 0.50. Thus, 46 students, who wished to participate in the study, were recruited after considering the dropout rate.

### 2.3. Data Collection and Ethical Considerations

Data were collected in April 2021. This study was approved by the Bioethics Committee of Gwangju University (IRB No. 2-1041318-A-A-01-202102-HR-004-01) for the ethical protection of the research participants. Students, who wished to participate, were informed regarding the study purpose and method a week before the scheduled KALS education session. In addition, they were also informed about the confidentiality and scientific use of the data collected, and their right to withdraw from the study at any time without any disadvantages. Those who provided written informed consent underwent a follow-up survey, after the KALS program, conducted by a qualified research assistant and researcher.

### 2.4. The Framework of KALS Training Program

The KALS training program was conducted twice for 6 h each (total of 12 h), in accordance with the KALS provider training program guidelines provided by the Korean Cardiopulmonary Resuscitation Association. Since Korean-style specialized professional resuscitation education should be conducted in teams, the attendees were divided into small groups of 5–6 people, and the program was implemented as shown in Table 1.

### 2.5. Research Instruments

#### 2.5.1. Korean Advanced Life Support Education on Non-Technical Skill Performance Ability

In this study, non-technical skill performance ability was assessed using the Team Emergency Assessment Measure (TEAM) Scale developed by Cooper et al. [15]. This tool comprises 11 items on leadership (2 items), teamwork (7 items), and work management (2 items), with a 5-point Likert scale (0 = *I* *never do*, 1 = *I don’t do it*, 2 = *I* *don’t do it often*, 3 = *I do it often*, 4 = *I* *always do*). The scores on this scale range from 0 to 44, where higher scores indicate higher non-technical skill performance. Cronbach’s α for this tool was 0.92 at the time of development [15], and 0.73 in this study.

#### 2.5.2. Communication Confidence

The standardized SBAR tool (situation, background, evaluation, and proposal) indicates the level of communication confidence in reporting a patient’s clinical situation to the medical staff. In this study, communication confidence was evaluated using the SBAR tool developed by Leonard et al. [16] and modified by Kim [17]. This tool comprises five items, with a numeric rating scale from 0 (*not* *at all confident*) to 10 (*very confident*). The scores on this scale range from 0 to 50, where higher scores indicate higher communication confidence levels. Cronbach’s α for this tool was 0.95 and 0.96, at the time of development and in this study, respectively.

#### 2.5.3. Critical Thinking Disposition

Nursing students’ critical thinking disposition was evaluated in this study using Yoon’s [18] Critical Thinking Disposition (YCTD) Instrument. This tool comprises 27 items, with a 5-point Likert scale ranging from 1 (strongly agree) to 5 (strongly disagree). The total scores on this scale range from 27 to 135, where higher scores indicate higher critical thinking propensity. Cronbach’s α for this tool was 0.84 in Yoon’s [18] study and 0.93 in this study.

#### 2.5.4. Self-Efficacy in Performing CPR

To measure self-efficacy in performing CPR, this study employed The Resuscitation Self-Efficacy Scale developed by Roh et al. [19] for evaluating individual ability to perform CPR. This tool comprises 12 items, with a 5-point Likert scale ranging from 1 (*not at all confident*) to 5 (*very confident*). The scores on this scale range from 12 to 60, where higher scores indicate higher self-efficacy in CPR performance. Cronbach’s α for this tool was 0.91 at the time of development and 0.83 in this study.

#### 2.5.5. Technical Skill Performance

The ability to perform specialized resuscitation in this study was evaluated using the Korean-type professional resuscitation instrument developed by the Korean Association of Cardiopulmonary Resuscitation (https://www.kacpr.org, accessed on 25 June 2021), in accordance with the advice of an emergency medicine specialist, with some modifications and supplements to suit this study. Technical skill performance evaluation was conducted by a nurse, who was in charge of CPR education, with more than 20 years of experience in BLS and KALS instructor training, and a nursing professor. In this study, the interobserver reliability of this tool was 0.86.

### 2.6. Statistical Analysis

Data analysis was performed using the SPSS WIN 25.0 program (IBM Corp., Armonk, NY, USA). The detailed analysis methods are as follows: (1) General participant characteristics were analyzed using frequency analysis and descriptive statistics. (2) Descriptive statistics, paired samples *t*-tests, and Pearson’s correlation coefficient were used to analyze the effects of the KALS program on non-technical skills, communication confidence, critical thinking tendency, self-efficacy in performing CPR, and technical skills. (3) The Cronbach’s α coefficient was used to evaluate the reliability of the tools. Statistical significance was set at *p* < 0.05.

## 3. Results

### 3.1. General Characteristics of the Participants

The average age of the current study participants was 22.23 ± 4.26 years, and all 46 participants were females. Of these, 33 (71.7%) and 13 (28.3%) were “satisfied” and “neutral” with their nursing major, indicating that most students were highly satisfied with their nursing major. In the past six semesters, 7 students (15.2%) obtained a grade of “A+~A”, while 34 (74.0%) and 5 (10.8%) students achieved “B+~B” and “C+~C”, respectively. All 46 participants underwent BLS training, of which 8 (17.4%) underwent training “6 months ago”, and 30 (65.2%) underwent training “12 months ago”, while the remaining 8 (17.4%) received training “18 months ago”, in the Department of Nursing at Shinsung University, Republic of Korea. BLS comprises a basic resuscitation curriculum, while KALS is a cardiac arrest simulation training course designed for emergency teams in the hospital. Furthermore, 34 (73.9%) participants stated that their reason for undergoing KALS training was “to obtain a certificate”, while for 12 (26.1%) others, it was “to apply for the desired department (special department)”. Table 2 presents the general characteristics of the participants. We believe that BLS certification (e.g., volunteer training, training as part of acquiring a driving license (as is the case in many countries)) did not influence the effectiveness of the KALS training.

### 3.2. Differences in Performance of Non-Technical Skills before and after KALS Training

The performance of non-technical skills was significantly higher after training than before (*t* = −5.892, *p* < 0.001). Table 3 presents the results of analyzing the differences in the domains of non-technical skill performance—leadership, teamwork, and work management. Teamwork (*t* = −6.563, *p* < 0.001) and work management (*t* = −6.415, *p* < 0.001) reported a statistically significant difference before and after the training, while leadership did not report a significant difference.

### 3.3. Differences in Communication Confidence, Critical Thinking Tendencies, and Self-Efficacy in Performing CPR before and after KALS Training

As shown in Table 4, communication confidence, critical thinking tendencies, and self-efficacy in performing CPR were found to be significantly higher after training than before (*t* = −6.563, *p* < 0.001; *t* = −3.352, *p* = 0.002; *t* = −13.242, *p* < 0.001, respectively).

### 3.4. Difference in Technical Skill Performance before and after KALS Training

The performance of technical skills was significantly higher after KALS training than before (*t* = −28.439, *p* < 0.001). As shown in Table 5, the results of analyzing the domains of technical skill performance report a significant difference in all domains—teamwork (*t* = −12.618, *p* < 0.001), ECG analysis (*t* = −25.984, *p* < 0.001), and management after cardiac arrest (*t* = −13.460, *p* < 0.001)—except for basic resuscitation.

## 4. Discussion

This study was conducted to support the evidence suggesting that resuscitation outcomes are affected by non-technical skills such as leadership and teamwork, since CPR is mainly performed in hospitals collectively rather than individually [20]. In this study, participants underwent an education program for 6 h in teams, following the KALS provider education program guidelines, prescribed by the Korean Cardiopulmonary Resuscitation Association. The results show that the participants’ non-technical skill performance, communication confidence, critical thinking tendencies, self-efficacy in performing CPR, and technical skill performance improved after obtaining Korean-style specialized resuscitation (KALS) education.

First, the teamwork and work management domains of non-technical skill performance reported significant differences before and after undergoing KALS training, but there was no significant difference in the leadership domain. The Korean Advanced Resuscitation Society specifies the elements of a successful resuscitation team: leadership, decision making, follow-up, and conflict resolution. Here, leadership refers to guiding team members for efficient task completion within a limited time through situation understanding, goal setting, role distribution, and feedback. However, specialized resuscitation is an unfamiliar and under-experienced situation for nursing students; it evokes embarrassment and tension and is a stressful situation for those lacking confidence in performing professional resuscitation. Therefore, performance of leadership roles is perceived to be a difficult task. Despite the limitations of the studies conducted on the non-technical skills of Korean advanced resuscitation education, a previous study [21] showed that the CPR performance scores were higher for the group with higher levels of non-technical skills than those with lower levels. In addition, the team leader’s non-technical skills are highly correlated with the CPR team’s technical performance [22]. Non-technical skills such as leadership can particularly minimize errors during CPR administration and improve patient outcomes [23]. Thus, in accordance with previous studies suggesting that acquisition of teamwork or leadership is relatively neglected, we found it necessary to provide non-technical skill education, including leadership, and examine its effect [24]. Moreover, despite the lack of correlation between the performance of well-defined professional resuscitation procedures and the general ability to perform non-technical skills, various external stressors may affect non-technical skills, such as leadership or communication, due to environmental factors. These environment stressors are of utmost importance amidst unpredictable emergencies [23,25]. However, this study did not account for negative stressors, such as those experienced by participants lacking clinical experience while administering advanced resuscitation; thus, future studies should consider these aspects.

Communication confidence reported significant improvements among study participants before and after receiving KALS education. This finding is consistent with previous study results indicating that team-based CPR training was effective for communication and cooperation among nursing students [26]. Thus, Korean-style specialized resuscitation training should be designed by organizing and operating teams of five to six people. This small-group team learning facilitates the process of free sharing and discussion among team members regarding the knowledge of the research participants about emergency situations. Therefore, it is presumed that communication confidence improved via problem solving and through mutual respect for their own and other participants’ opinions.

Furthermore, KALS education significantly improved the critical thinking tendencies of the current study participants. However, another study conducted with the Department of Emergency Rescue, using the same research tools, reported inconsistent findings with non-significant results for critical thinking tendency [27]. Critical thinking can be promoted through educational strategies by presenting students with multiple alternatives for achieving and verifying the most appropriate solution, rather than providing them with a unidimensional problem-solving method [28]. In addition, some argue that critical thinking can be promoted by providing data for analysis such as medical records, test data, and graphs of actual patients [29]. In this study, we provided debriefing during individual and team student practice and visualizations for various clues—from the four cardiac arrest cases—according to the changes in the patient’s condition, such as patient histories, ECG records, blood pressure, and blood test results. It is inferred that the learning strategy, which improved nursing students’ critical thinking tendencies, led to the expansion of thought processes and the selection of the most appropriate nursing intervention through constructive methods of collecting and analyzing various clues. Recently, several studies have been conducted on critical thinking tendencies due to the emphasis on its role in improving the analytic ability to make quick and accurate judgments about emergency patients within rapidly changing medical systems. However, the study results depicted differences depending on the training period, debriefing, clinical and simulation practice, practice time sufficiency, and opportunities for self-practice [27,30]. Moreover, few studies have directly examined the effects of KALS education on the critical thinking tendencies of nursing students. Further research needs to verify the effectiveness of applying various educational methods for KALS through repeated studies.

Similarly, the pretest–posttest results of examining self-efficacy in CPR report significant improvements among the current study participants after receiving the Korean-style specialized resuscitation (KALS) training. This finding is supported by previous studies on nursing college students and emergency rescue department college students [27,31,32]. However, unlike previous studies that used simulation-based CPR education for reporting effectiveness on variables such as self-efficacy, this study conducted KALS training through prior learning and the process of test, briefing, simulation, and debriefing. Our results also demonstrate the effect of implementing a simulated educational method similar to the clinical field, through a systematic education procedure. Thus, in addition to providing opportunities for individual prior learning and skill practice during training, this approach provides reinforcement by employing four cases of cardiac arrest, the process of recognizing clues for problem solving through repeated simulation practice, and debriefing through structured supportive and positive feedback provision on the simulation performed. Therefore, KALS education promotes a learner-centered process that recognizes and modifies its own mistakes and is considered to positively affect the motivation and self-efficacy of the participants in this study.

Lastly, technical skill performance reported significant improvements among the participants after the KALS training compared to before, but the BLS domain was not significant. BLS is a core basic nursing skill that involves the application of basic CPR and defibrillation, which is required to be performed by nursing students by their graduation year, in order to fulfill the certification standards prescribed by the Korea Institute of Nursing Education and Evaluation. For this purpose, educational institutions train students for a certain level of basic resuscitation, and most nursing students—looking for employment—acquire BLS certification before graduation. Therefore, the current study participants are also presumed to have educational experience of resuscitation, as BLS certification holders, before training in Korean-style specialized resuscitation.

Recently, the necessity of non-technical skills in CPR has been emphasized, and subsequently, the importance of non-technical skill education has gradually increased. This study is considered to be effective in improving the non-technical skills to cope with emergency situations for nursing students who want to acquire the Korean-style specialized resuscitation qualification as a prospective nurse. Simultaneously, the Korean specialized resuscitation curriculum plays an active role in clinical situations, considering that it is being implemented for doctors, dentists, nurses, first-class first responders, students in the final year of nursing and emergency medicine, etc. Therefore, it is necessary to ensure factors that facilitate active interaction in team activities, especially in the education of under-experienced nursing students, in order to enhance their emotional understanding of the situation.

However, this study had certain limitations. First, the study participants were recruited from a single university, and the sample size was small. Thus, the results of this study should be generalized cautiously. Second, this study is limited due to the lack of consideration for the effect of the stress level experienced by the participants amidst specialized resuscitation situations on non-technical and technical skill performance. Future studies must examine these effects on patient outcomes by simulating a structured and an unpredictable emergency, due to the interaction of bystanders, noise, and other distractions. Third, it is essential to verify the effects of the training period and time, debriefing, and provision of opportunities for self-practice in non-technical skills during professional resuscitation training. Fourth, non-technical and technical skill performance scores improved significantly. However, this study was unable to examine whether these differences reached the minimal threshold of clinical importance to improve the effectiveness of future BLS support. Moreover, the effect on critical thinking tendency must be verified in order to improve the analytic ability to judge emergency patients quickly and accurately in a rapidly changing medical system.

## 5. Conclusions

This study adopted a one-group pretest–posttest experimental design with 46 nursing students to examine the effects of KALS education on the non-technical skills and technical skills of nursing students. The results show that the participants’ scores for non-technical skill performance ability, communication confidence, critical thinking tendency, self-efficacy in CPR, and technical skill performance improved significantly after receiving KALS education. Accordingly, it is necessary to incorporate technical and non-technical skills in Korean-style specialized resuscitation education for nursing students.

## Figures and Tables

**Table 1:** KALS program contents.

KALS Program Contents	Method	Duration
Course summary, review of advanced life support, electrocardiogram rhythm with cardiac arrest	Lecture	1 h
Procedure lab and feedback Situation awareness and cardiopulmonary resuscitation team activation Chest compression and bag valve mask Alternative airway Defibrillation paddles, pads Emergency medication	Skill Practicum	1 h
Effective teamwork and leadership	Lecture	30 min
Simulation and debriefing session: cardiac arrest (4 cases) PEA, asystole, VF/VT session Decision making and task management Communication skills	Practicum	2 h
Electrocardiogram-guided KALS algorithm	Lecture	30 min
Non-technical and technical skill evaluation according to the cardiac arrest situation	Simulation Test	1 h

KALS: Korean Advanced Life Support; PEA: pulseless electrical activity; VF/VT: ventricular fibrillation/pulseless ventricular tachycardia.

**Table 2:** General participant characteristics (*n* = 46).

Characteristics	Categories	*n*	%
Age (years)	Mean ± standard deviation	22.23 ± 4.26	
Sex	Male	0	0
	Female	46	100
Satisfaction with major	Satisfied	33	71.7
	Neutral	13	28.3
	Dissatisfied	0	0
Grade	A+~A	7	15.2
	B+~B	34	74.0
	C+~C	5	10.8
Basic life support training	Yes	46	100
	No	0	0
Duration of basic life support training	6 months ago	8	17.4
	12 months ago	30	65.2
	18 months ago	8	17.4
Reasons for participation in resuscitation training	Certification	34	73.9
	Apply for desired (special) department	12	26.1

**Table 3:** Differences in non-technical skill scores before and after undergoing Korean Advanced Life Support training (*n* = 46).

		Pre	Post	*p*
Non-technical skills		30.58 ± 15.69	47.16 ± 4.41	<0.001
Domains	Leadership	5.84 ± 8.21	8.61 ± 0.95	0.062
	Teamwork	19.03 ± 8.21	29.74 ± 3.07	<0.001
	Work management	5.71 ± 2.56	8.81 ± 0.98	<0.001

Values are mean ± standard deviation. Tested by paired samples *t*-test.

**Table 4:** Communication confidence, critical thinking tendency, and self-efficacy in cardiopulmonary resuscitation before and after Korean Advanced Life Support training (*n* = 46).

	Pre	Post	*p*
Communication confidence	23.45 ± 8.70	35.77 ± 6.37	<0.001
Critical thinking tendency	96.71 ± 13.54	107.16 ± 11.93	0.002
Self-efficacy in performing cardiopulmonary resuscitation	33.56 ± 7.15	49.81 ± 5.61	<0.001

Values are mean ± standard deviation. Tested by paired samples *t*-test.

**Table 5:** Technical skill performance before and after Korean Advanced Life Support training (*n* = 46).

		Pre	Post	*p*
Technical skills		18.35 ± 2.07	27.94 ± 1.63	<0.001
Domains	Basic life support	3.84 ± 0.37	3.97 ± 0.18	0.103
	Mutual performance monitoring	6.74 ± 1.79	10.45 ± 1.31	<0.001
	Electrocardiogram analysis	5.56 ± 0.72	9.68 ± 0.54	<0.001
	Management after cardiac arrest	2.24 ± 0.72	3.84 ± 0.37	<0.001

Values are mean ± standard deviation. Tested by paired samples *t*-test.

## Data Availability

The data presented in this study are available on request to the authors.
